# GeoAI: Beyond mapping earth and cities through explainability, adaptability, and sustainability

**DOI:** 10.1016/j.isci.2025.114407

**Published:** 2025-12-26

**Authors:** Yongze Song, Filip Biljecki, Gustau Camps-Valls, Peter M. Atkinson

**Affiliations:** 1 School of Design and the Built Environment, Curtin University, Perth, WA, Australia; 2 Department of Architecture, National University of Singapore, Singapore, Singapore; 3 Department of Real Estate, National University of Singapore, Singapore, Singapore; 4 Image Processing Laboratory (IPL), Universitat de València, Valéncia, Spain; 5 Lancaster Environment Centre, Lancaster University, Bailrigg, Lancaster LA1 4YR, UK; 6 Geography and Environmental Science, University of Southampton, Highfield, Southampton SO17 1BJ, UK; 7 College of Surveying and Geo-Informatics, Tongji University, No.1239, Siping Road, Shanghai 200092, P.R. China

## Abstract

Geospatial artificial intelligence (GeoAI) is reshaping our understanding of Earth and urban systems by integrating advanced artificial intelligence techniques with diverse geospatial data and methodologies. This backstory highlights recent GeoAI advances and applications as presented in the 11 articles in the *iScience* special issue, “GeoAI shaping earth and cities: Advances, opportunities, and challenges.” Guest editors share perspectives on GeoAI’s advances in explainability, adaptability, and sustainability, demonstrating that GeoAI’s applications extend beyond traditional mapping functions. These 11 case studies illustrate four types of explainability, three levels of adaptability and three thematic areas of sustainability, showing the methodological diversity and practical relevance of GeoAI for Earth and urban systems. Here, interactions among these dimensions are mapped to support the evaluation and design of future GeoAI solutions. We also outline future research directions for GeoAI to address complex challenges across the sciences relating to the Earth and its cities.

**Figure:**
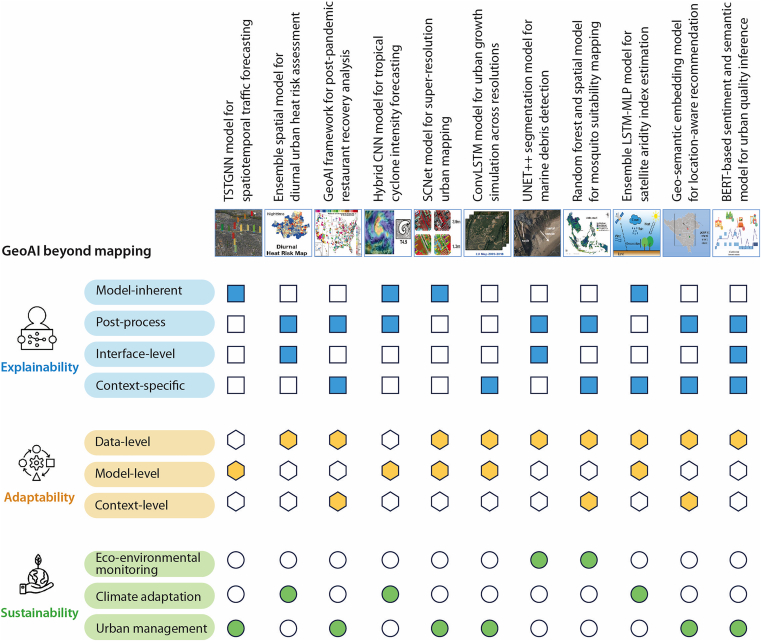
Above image: A conceptual framework illustrating how GeoAI advances beyond traditional mapping toward shaping Earth and city systems through the dimensions of explainability, adaptability, and sustainability The thumbnail images representing each case study are adopted from the figures in the corresponding articles published in this special issue.

Recent studies demonstrate how GeoAI extends beyond traditional mapping

GeoAI approaches can optimize resource use, reduce environmental risks and enhance societal well-being in ways that are both ecologically sound and socially responsible.

## Main text

Geospatial artificial intelligence (GeoAI) has emerged as a transformative paradigm at the intersection of artificial intelligence, geographic information science (GIS), and Earth observation, fundamentally reshaping how complex human-natural systems are understood and managed.^1^ With advances in machine learning, deep learning and high-performance computing, GeoAI can now integrate massive and heterogeneous geospatial datasets, including remote sensing, LiDAR, crowdsourced, and *in situ* data, for enhanced mapping, spatial prediction, and environmental monitoring across diverse areas.^2^ Recent studies have expanded GeoAI applications beyond traditional geospatial analysis to address dynamic urban systems, human geography, and complex socioenvironmental issues.^3^ At the same time, recent discussions point to a growing need for improved model explainability and adaptability across spatiotemporal contexts, long-term sustainability, scientific transparency, and societal impact of GeoAI.^4^ However, there remain essential gaps in domain-specific methodologies and practical applications for addressing real-world issues in broader fields of science relating to the Earth and its cities. In this backstory, guest editors of the iScience special issue GeoAI shaping earth and cities: Advances, opportunities, and challenges, critically discuss the current state and emerging directions of GeoAI research and examine how it can extend beyond conventional mapping functions to shape the future of Earth and urban systems through enhanced explainability, adaptability, and sustainability.

### GeoAI’s advances in explainability, adaptability, and sustainability for earth and its cities

GeoAI studies published in the recent iScience special issue demonstrate how GeoAI extends beyond traditional mapping. Figure 1 visualizes the general framework, illustrating how the three dimensions of *explainability, adaptability, and sustainability—*and their respective categories—are represented across the 11 case studies published in this special issue. The concepts, scope, and explanations of the cases are presented in the sections later.

#### Explainability

GeoAI approaches to explainability in addressing issues in the Earth and urban sciences can be classified into four categories: model-inherent, post-process, interface-level, and context-specific explainability.

The first approach, model-inherent explainability, includes model-level solutions such as explainable deep learning architectures, model simplification, and rule extraction. In these approaches, interpretability is inherently designed in attention-based or hybrid rule-based models. For example, the study using a hybrid convolutional neural network (CNN) model for tropical cyclone forecasting shows model-inherent explainability by embedding spatial-temporal features into the prediction architecture.^5^ This approach provides built-in interpretability aligned with physical cyclone dynamics for explainable deep learning in climate applications. The traffic forecasting study develops a temporal-spatial transformer graph neural network (TSTGNN) model by embedding spatiotemporal dependencies and heterogeneous traffic patterns into adaptive graph structures that interpret the learning process.^6^ The study on high-quality super-resolution mapping uses spatial deep learning through designing a deep convolutional neural network that integrates spatial context with fine-grained geographic details while enhancing resolution and accuracy.^7^

The second approach, post-process explainability, involves extracting information about model interpretation, case-based explanations, and local spatial analysis from the outcomes after model training. The commonly used methods for post-process explainability extraction include SHapley Additive exPlanations (SHAP), Local Interpretable Model-agnostic Explanations (LIME), and validation compared with benchmarks. For example, the sentence-bidirectional encoder representations from transformers (BERT)-based model for spatial-semantic recommendation analyses the correlation between semantic similarity and geographic proximity, and applies Moran’s *I* to interpret the spatial clustering of recommendation outputs, showing that evaluation of spatial-semantic alignment can reveal the underlying logic of deep learning-based geospatial recommendations.^8^ The GeoAI framework for post-pandemic restaurant recovery analysis applies entropy index, KS statistics, and gravity law models to interpret spatial patterns of restaurant recovery for the identification of mobility shifts, economic disparities, and localized resilience strategies, thereby providing practical targeted policy interventions across diverse urban regions.^9^ The urban sentiment analysis using the knowledge-based (KB)-BERT model uses case-based explanations and spatial visualization of semantic outputs across different city zones.^10^

The third approach involves interface-level explainability, which presents GeoAI model outcomes through visualization and interaction tools. Visualization and interaction tools include spatial distribution maps, overlays and web-based mapping platforms. For example, implementation of the U-Net convolutional neural network architecture (UNET++) segmentation model for marine debris detection visualizes model outputs as maps of coastal areas for user engagement and to support environmental monitoring through intuitive and interpretable spatial representations.^11^ The urban heat risk assessment using a stacking ensemble model uses fine-resolution spatial distribution maps and daily heat overlays to visually display risk zones, increasing the practical utility of model outputs for urban climate planning.^12^

The last approach is context-specific explainability, which demonstrates contextual strategies of GeoAI and modeling in specific fields, such as using context-aware explanations and validation using professional and expert knowledge. For example, in a study focused on mosquito suitability mapping, the random forest model is integrated with expert knowledge of ecological and climatic factors into model design and interpretation to ensure that the outputs align with vector ecology and public health decision-making.^13^ The convolutional long short-term memory network (ConvLSTM) model for urban growth simulation examines how different spatiotemporal resolution combinations affect urban growth simulation performance, where the ConvLSTM and comparative modeling provide context-aware guidance for resolution selection and to support evidence-based urban planning in fast-growing areas.^14^

#### Adaptability

In terms of adaptability, GeoAI approaches can be categorized into three types: data-level, model-level, and context-level adaptability.

Data-level adaptability refers to GeoAI’s ability to effectively deal with diverse and heterogeneous datasets that vary in source, resolution, type, or temporal frequency. This adaptability is critical when models need to integrate information from multiple sensors, geographic databases, or climate projections. For example, the TSTGNN model learns long-term dependencies and local variations from heterogeneous spatial-temporal traffic data for precise congestion forecasting and to increase prediction accuracy in dynamic urban systems.^6^ The ConvLSTM employs land use and environmental data at multiple spatiotemporal resolutions to increase prediction accuracy and robustness in simulating urban growth across fast-growing and stable areas.^14^ The UNET++ segmentation model incorporates refined label sets and fine-resolution multispectral Sentinel-2 inputs to increase marine debris detection accuracy in pixel-ambiguous coastal scenes.^11^

Model-level adaptability is the ability of GeoAI models to flexibly apply to different tasks, hazards, or contexts with minimal modification to, or tuning of, their underlying structure or parameters. The approaches usually involve designing reusable, interpretable, and capable-of-context-transfer architectures. For example, the hybrid CNN model integrating multi-scale features for tropical cyclone intensity forecasting increases accuracy and interpretability by enabling the model to focus dynamically on critical spatial-temporal patterns.^5^ The satellite aridity index estimation uses an ensemble LSTM-MLP model that can flexibly capture temporal dynamics and increase the accuracy of aridity index estimation across diverse climatic regions for drought monitoring accuracy and to support climate adaptation strategies in data-sparse areas.^15^

Context-level adaptability reveals the ability of GeoAI to generalize across geographic, environmental, or social contexts. Context-level adaptability emphasizes how well a model trained in one region or setting can perform in another. For example, the random forest model generates a 500 m spatial resolution mosquito suitability map across diverse climatic zones in Southeast Asia, benefiting vector control by capturing spatial variability in environmental conditions.^13^ A GIS-based model analyzing restaurant recovery after COVID-19 captures divergent economic rebound patterns across US cities, providing localized economic conditions by adapting to socioeconomic and spatial heterogeneity.^9^

#### Sustainability

GeoAI for sustainability means applying models to support long-term, balanced development by promoting environmental resilience, urban liveability, and climate adaptability through efficient monitoring, forecasting, and decision-making. GeoAI approaches can optimize resource use, reduce environmental risks, and enhance societal well-being in ways that are both ecologically sound and socially responsible. In this special issue, the sustainability issues addressed by GeoAI can be classified into eco-environmental monitoring, climate adaptation, and urban management.

From the perspective of eco-environmental monitoring, a random forest-based species distribution model combined with spatial autocorrelation and geographical detectors is to map long-term mosquito habitat suitability, providing fine-resolution analysis of vector-borne disease risks and supporting targeted environmental health interventions across Southeast Asia.^13^ In addition, GeoAI enables accurate, large-scale detection of marine debris using deep learning segmentation models on fine-resolution imagery, which supports efficient monitoring of coastal ecosystems and targeted cleanup efforts. In this case, the developed model makes environmental monitoring more responsive and cost-effective through handling noisy data, learning contextual spatial features, and scaling detection.^11^

From the perspective of climate adaptation, the study on aridity analysis not only reveals regional drying trends through fine-resolution satellite-derived aridity indices, but also enables dynamic, forward-looking water management decisions through its ability to learn and update continuously from evolving multi-temporal climate data.^15^ In addition, GeoAI enhances climate-resilient urban planning by using ensemble machine learning to generate fine-resolution urban heat risk maps that reflect daily and seasonal climate variability, guiding interventions in cities facing intensifying heat extremes to determine real-world heat vulnerability.^12^

From the perspective of urban management, urban growth, simulated using ConvLSTM, demonstrates how optimizing spatiotemporal input resolutions can increase prediction accuracy in fast-developing cities for infrastructure planning and resilient urban expansion strategies.^14^ The urban sentiment and semantic analysis using the KB-BERT model contributes to socially informed urban planning by mapping public perceptions and emotional responses across districts.^10^ The traffic forecasting using a TSTGNN model supports intelligent urban infrastructure management by capturing long-term and localized traffic patterns for proactive congestion mitigation and transportation planning in urban networks.^6^

### Future directions

Future research on GeoAI for studying the Earth and its cities should advance in the following directions. First, improving data quality, diversity, granularity, and source integration is essential. Enhancing data quality includes incorporating a broader range of vulnerability indicators, extending temporal records using next-generation satellite products, adopting finer-resolution environmental data, and expanding annotated datasets through expert-informed labeling. Second, there is an urgent need for methodological development and model optimization. Methodological development includes developing more robust classification and unmixing algorithms, implementing phase-specific hybrid deep learning models and increasing computational efficiency in transformer- and graph-based frameworks. Third, GeoAI methods should be applied in real-world validation and practical applications, such as applying models across diverse, representative datasets and fine-resolution satellite sensor imagery, and validating predictions with ground reference or crowdsourced data. Finally, contextual and interpretative enrichment is required for domain-specific applications. Potential approaches include integrating socio-cultural factors, addressing scale-dependent modeling challenges and enhancing interpretability through spatial-semantic mapping and expert-driven assessment.

## Acknowledgments

The guest editors extend their sincere gratitude to all contributing authors and dedicated reviewers for their invaluable contributions to this special issue. We would also like to sincerely thank Ryan Perry for his essential support and efforts in editing the special issue.

## Declaration of interests

The authors declare no competing interests.
